# Isolation and Characterization of a Phosphate-Solubilizing Halophilic Bacterium *Kushneria* sp. YCWA18 from Daqiao Saltern on the Coast of Yellow Sea of China

**DOI:** 10.1155/2011/615032

**Published:** 2011-06-02

**Authors:** Fengling Zhu, Lingyun Qu, Xuguang Hong, Xiuqin Sun

**Affiliations:** First Institute of Oceanography, State Oceanic Administration of China, No. 6 Xianxialing Road, High-Tech District, Qingdao 266061, China

## Abstract

Phosphate-solubilizing bacteria (PSB) function in soil phosphorus cycle, increasing the bioavailability of soil phosphorus for plants. Isolation and application of salt-tolerant or halophilic PSB will facilitate the development of saline-alkali soil-based agriculture. A moderately halophilic bacterium was isolated from the sediment of Daqiao saltern on the eastern coast of China, which also performs phosphate-solubilizing ability. The bacterium was assigned to genus *Kushneria* according to its 16S rRNA gene sequence, and accordingly named as *Kushneria* sp. YCWA18. The fastest growth was observed when the culturing temperature was 28°C and the concentration of NaCl was 6% (w/v). It was founds that the bacterium can survive at a concentration of NaCl up to 20%. At the optimum condition, the bacterium solubilized 283.16 **μ**g/mL phosphorus in 11 days after being inoculated in 200 mL Ca_3_(PO_4_)_2_ containing liquid medium, and 47.52 **μ**g/mL phosphorus in 8 days after being inoculated in 200 mL lecithin-containing liquid medium. The growth of the bacterium was concomitant with a significant decrease of acidity of the medium.

## 1. Introduction

Phosphorus (P) is one of the major essential macronutrients for plants, which is applied to the soil in the form of phosphatic manure. However, a large portion of the applied phosphorus is rapidly immobilized, being unavailable to plants [1]. In average, the content of phosphorus of soil is about 0.05% (w/w); however, only 0.1% of them are usable for plants [2]. Saline-alkali soil-based agriculture develops quickly in recent years. Similar to the fertile soil-based agriculture, the intensive culturing of salt-tolerant and even salt-resistant plants has dramatically decreased the availability of phosphorus in saline-alkali soil. The free phosphatic ion in soil plays a crucial role; the orthophosphatic ion is the only ion which can be assimilated in an appreciable amount by plants [3]. Soil microorganisms involve in a wide range of biological processes including the transformation of soil phosphorus. They solubilize soil phosphorus for the growth of plants [4].

The growth of phosphate-solubilizing bacteria (PSB) often causes soil acidification, playing a key role in phosphorus solubilization [5]. Therefore, PSB are considered the important solubilizers of insoluble inorganic phosphate. In turn, plants reimburse PSB with carbohydrates [6]. Since the beginning of last century, many PSB have been isolated including, for example, those in *Bacillus*, *Pseudomonas*,* Erwinia*, *Agrobacterium, Serratia*, *Flavobacterium*, *Enterobacter*,* Micrococcus*, *Azotobacter*, *Bradyrhizobium*, *Salmonella*,* Alcaligenes*, *Chromobacterium*, *Arthrobacter*, *Streptomyces*, *Thiobacillus, *and *Escherichia* [7]. The microorganisms functioning similarly also include some fungi in genus *Penicillium*, *Aspergillus*,* Rhizopus*, *Fusarium, *and *Sclerotium* [7]. Unfortunately, most PSB isolated previously performed relatively low salinity tolerance, being less appropriate for saline-alkali soil-based agriculture. It is urgently needed to isolate highly halophilic PSB for the development of saline-alkali soil-based agriculture. In this study, a moderately halophilic, phosphate-solubilizing bacterium YCWA18 was isolated and characterized.

## 2. Materials and Methods

### 2.1. Bacterial Isolation

The sediment was sampled at Daqiao saltern, Jimo, Qingdao, on the eastern coast of China (E 120°49′12′′, N 36°30′00′′). Approximately 1 g sediment was suspended in 100 mL sterilized seawater and vortexed for 10 min. The isolates were obtained by plating a serial of 10-fold dilutions of sediment suspension onto a modified marine agar medium (2216E, one liter of seawater contains 5 g tryptone, 1 g yeast extract and 15 g agar, pH 7.5) [8] and incubating at 28°C for 7 days. The isolates were purified by restreaking on 2216E agar plate and microscopic confirmation.

The isolates were screened for their phosphorus-solubilizing ability by culturing at 28°C on the media supplemented either lecithin or Ca_3_(PO_4_)_2_. When the colonies appeared in one week, those causing a clear phosphate-solubilizing zone were selected out for further characterization. The size of phosphate-solubilizing zone was determined for each colony.

The modified Ca_3_(PO_4_)_2_ culture medium contained with the following ingredients (l^−1^) [9]: glucose 10 g, (NH_4_)_2_SO_4_ 0.5 g, NaCl 30 g, KCl 0.3 g, FeSO_4_·7H_2_O 0.03 g, MnSO_4_·4H_2_O 0.03 g, MgSO_4_·7H_2_O 0.3 g, Ca_3_(PO_4_)_2_ 10 g, agar 20 g, H_2_0 1000 mL, pH 7.0–7.5. The lecithin culture medium was composed of (l^−1^) [10]: glucose 10 g, (NH_4_)_2_SO_4_ 0.5 g, NaCl 30 g, KCl 0.3 g, FeSO_4_·7H_2_O 0.03 g, MnSO_4_·4H_2_O 0.03 g, MgSO_4_·7H_2_O 0.3 g, lecithin 0.2 g, CaCO_3_ 5 g, yeast extract 0.4 g, agar 20 g, H_2_0 1000 mL, pH 7.0–7.5.

The ingredients were prepared and sterilized by an autoclave for 20 min at 115°C without lecithin. Lecithin was prepared by diluting in sterile water and was added to the medium before inoculation. 

Bacterial isolate was freeze-stored in 2216E medium supplemented with 30% (v/v) glycerol at −80°C.

### 2.2. Bacterial Characterization

The isolate was phenotypically characterized following the minimal standards for describing the new taxa of family *Halomonadaceae* recommended previously [11]. Gram-staining was carried out with the method described by Dussault [12]. Anaerobic growth performance was determined by inoculating the semisolid 2216E (0.6% agar, w/v) at the bottom of tube and sealing with 2 mL agar (2%, w/v) and 2 mL paraffin. Acidification in liquid medium was observed using bromocresol purple supplemented with 1% of carbohydrate. Other morphological, physiological, and biochemical characterizations were done as described by Mata et al. [13].

### 2.3. Tests of Salt, pH, and Temperature Tolerance

NaCl tolerance was determined in 2216E medium containing 0, 0.5, 1, 2, 3, 4, 5, 6, 7, 8, 9, 10, 12.5, 15, 20, 25, and 28% (w/v) total salts. The pH range for growth was tested at pH 2.0–11.0 in increments of pH 1.0. Growth was determined at A_600_. Growth at 0, 6, 10, 15, 20, 24, 28, 32, 37, 42, and 45°C was also determined.

### 2.4. Taxonomical Assignment

DNA was extracted with the method of Hiraishi [14] and used as the template for the amplification of 16S rRNA gene with universal primers 27F and 1492R [15]. The sequence obtained was aligned with its orthologs retrieved from both GenBank and TYP16S databases. The phylogenetic assignment was performed using MEGA software with bootstrap percentages calculated with 1000 replications.

### 2.5. Determination of Phosphorus-Solubilizing Ability

The bacterium was inoculated into 200 mL liquid media supplemented with either Ca_3_(PO_4_)_2_ or lecithin and cultured at 28°C for 12 days with continuous agitation (150 r/min). 10 ml culture was sampled aseptically every 24 hours for the determination of acidity and available phosphorus. The acidity was assayed simply by reading on a pH meter, and the phosphorus availability was determined with Mo-blue method [16]. Optimum pH and temperature for P-solubilization in liquid Ca_3_(PO_4_)_2_ medium were determined following the above method.

## 3. Results

### 3.1. Characterization of the Isolate

**Figure 1:**
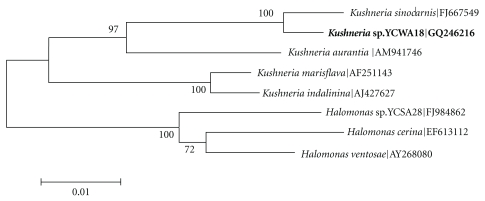
Neighbor-joining phylogenetic tree based on 16S rRNA gene sequences, showing the position of strain YCWA18 with respect to related species.

**Figure 2:**
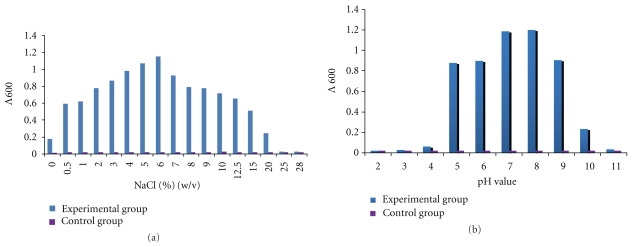
Test of NaCl and pH tolerance of the isolate ((a): NaCl; (b): pH).

The closest species of the isolate was* Kushneria sinocarnis*; the similarity between their 16S rRNA genes is 98.57%. As shown in Figure 1, the isolate was assigned to the clade of *K. sinocarnis* with 100% bootstrap support. The isolate was named *Kushneria* sp. YCWA18. It is a Gram-negative bacterium and grows aerobically. Its colony is yellow and round with a diameter between 1.2–2.5 mm after growing on 2216E for 48 hours. The fastest growth is observed at 6% NaCl (w/v), 28°C and pH 7.0–8.0 (Figure 2). The isolate can survive at high concentrations of NaCl (up to 20%) and pH range of 4.0–10.0 (Figure 2). It reduces nitrate and hydrolyzes aesculin but not urea, Tween20 and Tween80. The isolate produces acid from carbohydrates including D-fructose, *α*-D-glucose, D-mannose, maltose, D-sorbitol, succinamic acid, adonitol and L-alanyl-glycine but not D-psicose, D- glucosaminic acid, and inosine.

### 3.2. Growth on Ca_3_(PO_4_)_2_ and Lecithin Containing Solid Media

**Figure 3:**
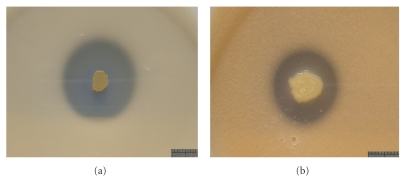
The phosphate-solubilizing zone formed on medium containing Ca_3_(PO_4_)_2_ (a) and lecithin (b). Bar: 1 cm.

The isolate grows well at 28°C on lecithin and Ca_3_(PO_4_)_2_ containing solid media. In 3 days (lecithin containing medium) or 5 days (Ca_3_(PO_4_)_2_ containing medium), clear phosphate-solubilizing zone forms. In 10 days, the phosphate-solubilizing zone expanded to the biggest (about 2.5–3.0 cm on Ca_3_(PO_4_)_2_ containing plate, Figure 3(a) and about 1.7–2.0 cm on lecithin containing medium, Figure 3(b)).

### 3.3. Growth in Ca_3_(PO_4_)_2_ and Lecithin-Containing Liquid Media

**Figure 4:**
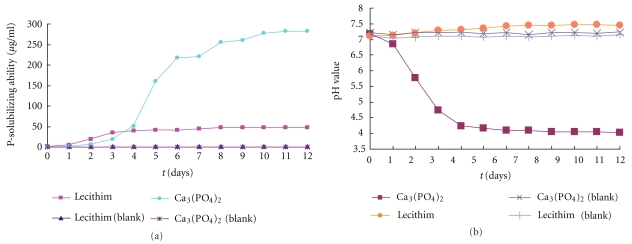
Phosphorus-solubilizing performance of the isolate ((a) P-solubilizing ability; (b) pH value of the medium).

As showed in Figure 4, Ca_3_(PO_4_)_2_ solubilization is slow in the first two days and then becomes fast, reaching the highest (283.16 *μ*g/mL) in about 11 days. Lecithin solubilization starts to increases in 1 day, reaching the highest 47.52 *μ*g/mL in 8 days. It was found that the growth of the isolate caused a significant increase of acidity in Ca_3_(PO_4_)_2_ containing medium. In about 4 days, the acidity increased from pH 7.21 to pH 4.24. In contrast, the acidity decreased from pH 7.1 to pH 7.46 in lecithin-containing medium.

### 3.4. The Influence of Temperature and Acidity on Phosphorus Solubilization in Ca_3_(PO_4_)_2_-Containing Medium

**Figure 5:**
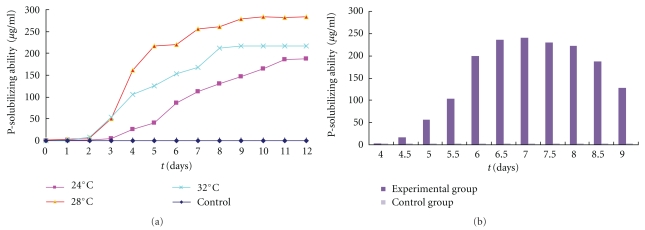
The influence of temperature (a) and acidity (b) on the phosphorus solubilization in Ca_3_(PO_4_)_2_ containing medium.

As showed in Figure 5, the concentration of soluble phosphorus in Ca_3_(PO_4_)_2_ containing medium starts to increases in 2 days when the isolate was cultured at 28°C and 32°C, reaching the highest in 7-8 days, faster than the increment achieved at 24°C. At 28°C, the bacterium obtained the highest solubilizing ability of Ca_3_(PO_4_)_2_, about 283.16 *μ*g/mL in 200 mL Ca_3_(PO_4_)_2_ containing medium at 28°C, while at 32°C and 24°C, it obtained the ability of 217.58 *μ*g/mL, and 187 *μ*g/mL, respectively, and it was found that phosphorus solubilization reached the maximum when the pH value of Ca_3_(PO_4_)_2_ containing medium is 7.0. 

## 4. Discussions

Phosphorus is an important limiting factor in agriculture production, and microbial activation seems to be an effective way to solve the solidified phosphorus in soil. Many bacterial strains with P-solubilizing abilities have been examined in previous studies, but few of them can function well at high NaCl concentration, and reports on isolation of halophilic PSB strain have not been found yet. The result obtained in this study shows that the isolate YCWA18 has a broad growth range: it can survive at high concentrations of NaCl (up to 20%) and pH range of 4.0–10.0, and it can solubilize both inorganic phosphorus and organophosphorus, and result also shows that its P-solubility for Ca_3_(PO_4_)_2_ is higher than for lecithin (Figure 3).

The formation of the clear zones is concerned with the P-solubilization of the strain (Figure 3). It may secrete some substances into surroundings in the course of growing, which can solubilize phosphate or organophosphate. P-solubilization result may vary depending on kinds of the metabolin, how quickly it releases, and also its spread degree on the medium. Therefore, observational method of P-solubilizing zone can only be used to qualitative assays [17].

Phosphorus solubilization is a complex process, which is influenced by diverse factors such as nutritional richness and physiological and growing status of the bacterium [18]. A number of theories have been proposed to explain the mechanism of phosphate solubilization, and the most important among them are acid production theory and enzyme theory. According to the acid production, the process of phosphate solubilization by PSB is due to the production of low molecular weight organic acids that was accompanied by the acidification of the medium [6, 19, 20], and those organic acids can chelate the cation with their hydroxyl and carboxyl groups [21]. The analysis of culture filtrates of PSBs has shown the presence of number of organic acids such as malic, glyoxalic, succinic, fumaric, tartaric, alpha keto butyric, oxalic, citric, 2-ketogluconic, and gluconic acid [22–24]. A decrease in the pH of the medium from the initial value of 7.0 to a final value of 2.0 was recorded by many workers [25, 26], and in our study, the decrease of the pH of Ca_3_(PO_4_)_2_-containing medium from pH 7.21 to pH 4.24 also supported this theory.

Enzymolysis is the possible mechanism of lecithin solubilization [27]. The acidity of lecithin-containing medium during culturing is a little higher than that of the control (Figure 4(b)). We believe that phosphorus solubilization from lecithin is not through acidification. It is possible that the increase of lecithin-containing medium during culturing is caused by enzymes which act on lecithin and produce choline.

The association among bacterial growth, supernatant acidity, and the concentration of solubilized phosphorus has been found [28]. As a well-developed strategy, microorganisms have been recruited to transform insoluble phosphorus of mineral sources into the soluble one [29]. As a particular case, Gram-negative bacteria mobilize insoluble phosphate very efficiently; they produce gluconic acid during the extracellular oxidation of glucose catalyzed by quinoprotein glucose dehydrogenase [30]. Microbial phosphorus mobilization should be the only way of increasing plant-available phosphorus in soil [31]. 

Diverse microorganisms are able to change phosphorus forms; however, their transforming ability may associate with the ecological conditions including soil characteristics and vegetation. It has been found that the performance of phosphorus-solubilizing microorganisms is severely affected by environmental factor, especially stress ones [32, 33]. In order to obtain the bacteria with high phosphorus-solubilizing ability, it is important to use samples of environmental extremes, for example, saline-alkali soil. In this work, a high phosphorus-solubilizing bacterium was isolated from the sediment if a saltern. It can grow on the solid media containing 20% (w/v) of sodium chloride, being halotolerant according to the suggested standards [34]. The biological characteristics and factors influencing its phosphate-solubilizing ability need to be studied further. Our trials paved an effective avenue of isolating halophilic and phosphorus-solubilizing bacteria from marine environment in order to promote saline-alkali soil-based agriculture.
